# Aggressive Inflammatory Myofibroblastic Tumor of Distal Pancreas: A Diagnostic and Surgical Challenge

**DOI:** 10.7759/cureus.22820

**Published:** 2022-03-03

**Authors:** Harisankar A G, Saket Kumar, Saurabh Singla, Nishant Kurian

**Affiliations:** 1 Department of Surgical Gastroenterology, Indira Gandhi Institute Of Medical Sciences, Patna, IND; 2 Department of Surgical Gastroenterology, Indira Gandhi Institute of Medical Sciences, Patna, IND; 3 Department Of Surgical Gastroenterology, Indira Gandhi Institute Of Medical Sciences, Patna, IND

**Keywords:** multivisceral resection, immunohistochemistry, distal pancreaticosplenectomy, gastrointestinal stromal tumor, inflammatory myofibroblastic tumor

## Abstract

An inflammatory myofibroblastic tumor (IMT) is a rare soft tissue neoplasm of unknown etiology. It is a slow-growing tumor of borderline malignant potential. Distant metastases and recurrence after complete excision are rare. Establishing a preoperative diagnosis is difficult because of its nonspecific clinic-radiological features. Although the majority of cases have been reported in the lungs, it can affect any part of the body. The pancreatic inflammatory myofibroblastic tumor is very rare and only 26 cases have been reported in the medical literature. These tumors mostly arise from the head of the pancreas, whereas occurrence in the body or tail region is rather unusual. Here, we report a case of a 55-year-old male patient with a locally advanced inflammatory myofibroblastic tumor arising from the pancreatic tail. Complete excision of tumor required multi-visceral resection (distal pancreaticosplenectomy with jejunal and colonic segmental resection). The diagnosis of inflammatory myofibroblast tumor was made on the basis of histopathology and immunohistochemistry.

## Introduction

The term inflammatory myofibroblastic tumor was first proposed in 1990 by Pettinato et al. based on their study on 20 cases of inflammatory tumors of the lung and esophagus [1]. According to the current World Health Organisation (WHO) guidelines, Inflammatory myofibroblastic tumors (IMT) are typically low-grade neoplasms with occasional malignant potential [2]. It is mostly reported in children and young adults but can affect people of any age. It is frequently reported in the lungs, abdomen, retroperitoneum, and limbs [3]. Pancreatic IMT is exceptionally rare and poses a diagnostic dilemma due to its vague clinical and radiological manifestations. Diagnosis is typically established postoperatively on histopathology. Radical excision is usually curative, but rare cases of local recurrence and metastatic forms have also been reported [4]. Here, we report a rare case of distal pancreatic IMT with extensive local infiltration in a middle-aged man that necessitated multivisceral resection for complete excision.

## Case presentation

A 55-years-old male patient presented with a history of intermittent left upper abdominal pain for three months. The pain was associated with nausea and occasional vomiting that typically occurred 2-5 hours after food intake. He was initially evaluated and prescribed medical treatment at a local hospital but got no effective relief. Because of the nonspecific nature of complaints, he was initially not evaluated any further. However, his symptoms progressively worsened over the last one month, and at presentation, he had bilious vomiting two to three times a day. He also had anorexia and significant loss of weight over the past six months, but no jaundice, melena, or fever. The patient’s family history was not significant. The patient also revealed a habit of tobacco chewing for the last 15 years. General examination was unremarkable except for severe pallor. Abdominal examination revealed a large, well-defined, non-tender, mobile mass in the left hypochondrium, extending into the epigastrium and left iliac fossa. Blood investigation revealed hemoglobin 6.5 mg/dl, total leucocyte count- 14.47cu/mm. Other blood parameters and tumor markers (carcinoembryonic antigen (CEA) and cancer antigen 19-9 (CA19-9)) levels were within the normal range.

**Figure 1:**
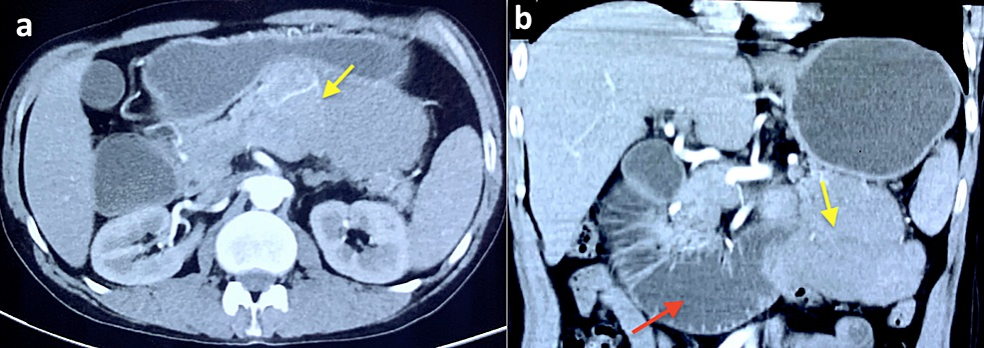
Contrast-enhanced computed tomography images (a) Axial sections showing heterogeneously enhancing lobulated lesion *(arrow)* involving tail of pancreas (b) Coronal sections showing infiltration of jejunum by the tumor *(yellow arrow) *causing upstream dilation of the proximal bowel loops *(red arrow).*

Contrast-enhanced computed tomography (CECT) scan of the abdomen was obtained that revealed a large, lobulated, well-defined, heterogeneously enhancing mass measuring approximately 11x8.0 cm arising from the body-tail of the pancreas. Multiple internal necrotic areas were seen with no obvious calcification. The tumor was infiltrating the jejunal loop with marked dilation of the proximal jejunal and duodenum (Figure1, panel a). The splenic artery and vein were completely encased by the tumor. The lesion was also abutting the distal transverse colon without any luminal compromise (Figure 1, panel b).

Based on clinical and radiological features, a provisional diagnosis of pancreatic or proximal jejunum gastrointestinal stromal tumor (GIST) was made. The patient was optimized with packed red blood cell transfusions and was planned for complete resection of the tumor. On exploration, a hard mass of approximately 15x10cm arising from the distal pancreas was found. The tumor was completely encasing the splenic artery. A loop of jejunum approximately 10cm from the duodenojejunal junction along with a segment of the transverse colon were found infiltrated by the tumor. The duodenum and proximal jejunal segment were dilated. To achieve complete tumor resection, distal pancreaticosplenectomy with segmental resection of the jejunum and transverse colon was performed, and the specimen was sent for histopathological examination. Reconstruction required two anastomoses. First, a side-to-side duodenojejunal anastomosis was done in two layers, followed by end-to-end colo-colic anastomosis in a single layer. Postoperative recovery was unremarkable except for Grade A pancreatic leak. The patient was discharged on a postoperative day eight with the abdominal drain in situ. The drain was removed during the outpatient department (OPD) visit after two weeks. The patient remained well in follow-up till three months of surgery.

**Figure 2:**
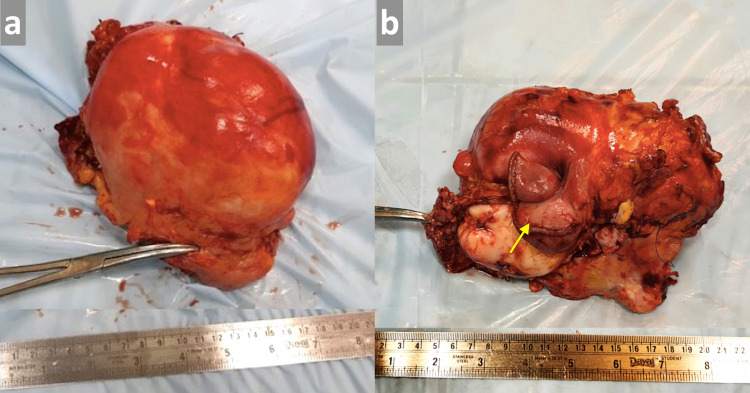
Resected tumor specimen: gross features (a) A resected specimen showing well-encapsulated neoplasm; (b) tumor with attached part of the infiltrated jejunal segment

Grossly, the tumor was fairly encapsulated, grey-white in color, and measured approximately 10x15cm in size. The tumor was infiltrating a segment of the proximal jejunum (Figure 2, panels a, b) as well as the transverse colon. 

**Figure 3:**
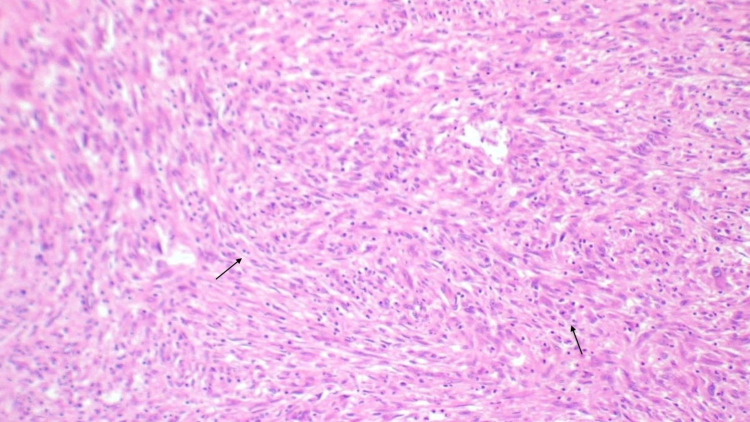
Histopathological image (magnification 40X) Hematoxylin and Eosin staining of the lesion showing fascicles of the spindle to plump cells exhibiting moderate nuclear pleomorphism. Few mononuclear and multinucleated giant cells were also seen. Intervening stroma shows dense chronic inflammatory cells mainly plasma cells and lymphocytes.

The histopathological examination revealed a fairly circumscribed tumor composed of fascicles of spindle cells to plump cells exhibiting moderate nuclear pleomorphism (Figure 3). 

**Figure 4:**
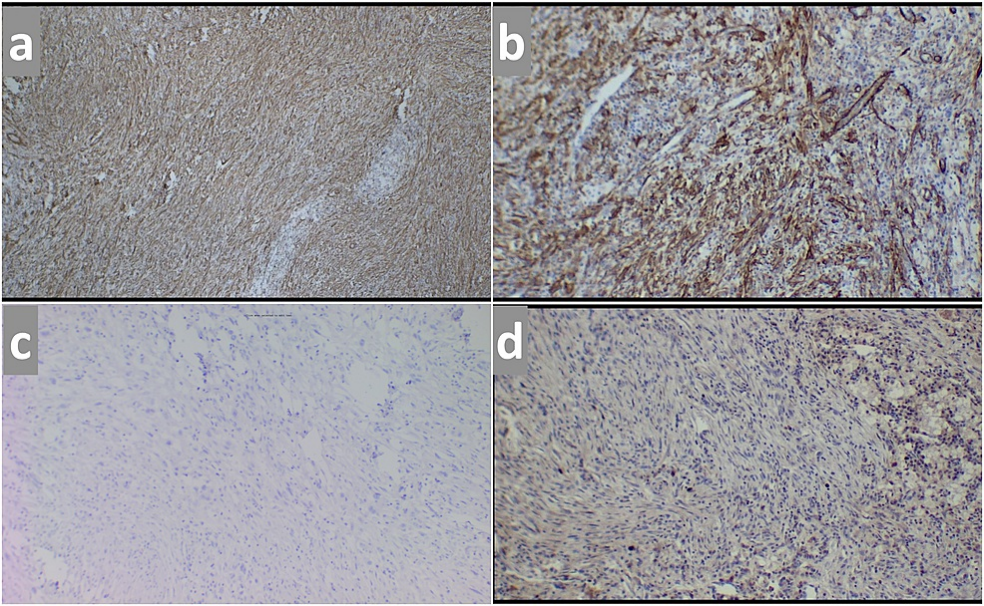
Immunohistochemistry images (magnification 40X) Tumor cells (a) positive for smooth muscle actin (SMA); (b) positive for Caldesmon; (c) negative for Anaplastic Lymphoma Kinase (ALK); (d) expressing Ki67 index of 10%.

Spindle cells showed mild to moderate atypia. Few mononuclear and multinucleate tumor giant cells were seen. Intervening stroma showed dense chronic inflammatory infiltrate comprising of plasma cells and lymphocytes. Resection margins were free of tumor invasion (R0 resection). Resected jejunum showed serosal tumor infiltration, however, the mucosa was uninvolved. The adherent colonic segment showed no infiltration by the tumor cells. Immunohistochemistry (IHC) report showed positive staining of tumor cells for Smooth muscle antigen (SMA) and caldesmon and negative for Anaplastic Lymphoma Kinase (ALK), S100, Synaptophysin, CD 34, CD 117, and DOG1. Ki 67 index was 10% (Figure 4, panels a, b, c, d).

On the basis of histopathology and IHC markers, a diagnosis of pancreatic IMT was made.

## Discussion

IMT is a rare neoplasm of unknown etiopathogenesis. It has been referred to with various synonyms in the medical literature such as plasma cell granuloma, xanthomatous pseudotumor, xanthoma, fibrous xanthoma, histiocytoma, xanthogranuloma, inflammatory myofibroblastic tumor, inflammatory myofibrohistiocytic proliferation, inflammatory pseudotumor, etc. Much clarity about this entity was obtained in the recent WHO classification (5th edition, 2020) of soft tissue tumors, where IMT was described as a separate entity, comprising of myofibroblastic and fibroblastic spindle cells with stromal inflammatory infiltrate [2]. The natural history, clinical course, and prognosis of these tumors are highly diverse, ranging from being totally benign to highly aggressive with fatal outcomes.

The IMT can arise from any part of the body and has usually been reported in children and young adults [2]. Nonaka et al. (2005), in a literature review of 730 IMT cases, reported the mean age of occurrence to be 29.6 years, with a wide range of 0 to 87 years. No gender predilection was found, and the most frequent site was the lungs [5].

A recent literature review by Matsyubayashi et al. (2019) included 28 patients of IMT occurring in the pancreas [6]. The reported mean age was 40 years (range, 0-82 years) with slight male preponderance. In the majority (71%), the tumor was located in the head of the pancreas. In this series, abdominal pain was the most frequent symptom followed by jaundice [6].

Similarly, we performed a literature survey of all published cases of pancreatic IMT in adults till December 2021. We didn't include pediatric patients or cases without histopathological proof of IMT. A total of 26 cases of pancreatic IMT in adults were found. The average age of presentation was 54.2 years (range, 15-82 years) with strong male predilection (~65.5%). The majority of the cases were located in the head region (54%) (14), while 23% (6) were located in the tail, 7% (2) in the neck-body of the pancreas, and only one case involved the body-tail region. The mean tumor size was approximately 5 cm (range, 1.5-10cm). Abdominal pain was the most common symptom (56%), followed by jaundice (36%) and loss of weight (24%). One patient had a recurrence in the form of pulmonary metastasis, whereas another patient died due to sepsis. No recurrence was reported in the remaining 24 patients in the follow-up. Only three cases of pancreatic IMT have been reported with colonic infiltration in the medical literature [7-10]. In our patient, the tumor was located in the body-tail region of the pancreas and was infiltrating into the jejunum and colon. He also had manifestations of proximal small bowel obstruction due to jejunal infiltration.

Our patient had presented with leukocytosis and severe anemia. There was no obvious gastrointestinal bleed. Although the tumor was infiltrating the jejunum, it was not eroding its mucosa. So, the anemia in our patient could have resulted from nutritional factors are because of the paraneoplastic effect of the tumor itself. Few previously published articles have reported an association of IMT with unexplained anemia and leukocytosis [11-12]. 

Preoperative diagnosis of IMT is difficult due to non-specific clinicoradiological features. With reference to previously reported cases, imaging findings may vary from non-enhancing to heterogeneously enhancing patterns on CECT scan. The lesion may appear small, well-circumscribed on one extreme, to bulky lobulated mass with extensive local infiltration on the other extreme [13]. Our patient had a large, heterogeneously enhancing tumor with central necrosis infiltrating into the adjacent jejunal loop and colon. Based on the imaging findings, a provisional diagnosis of GIST or adenocarcinoma of the distal pancreas was made. 

The final diagnosis of IMT is usually based on pathological examination. The tumor grossly appears as a well-circumscribed lobular, multinodular, or whorled mass of white or tan color with a fleshy or myxoid appearance. The tumor size may vary from as small as 4mm to as big as 36cm. Microscopically, IMT is composed of spindle cells with stromal inflammatory infiltrates. Higher mitotic phase and atypia of the spindle cells have been associated with increased chances of recurrence, invasion, and metastasis. On the basis of stromal pattern, three histological variants have been described, namely myxoid, hypercellular pattern (compact spindle cell pattern), and hypocellular fibrous pattern [14]. Mild to moderate cellular atypia can usually be seen, whereas spindle or atypical oval cells feature occasionally. These spindle cells often show anaplastic lymphoma kinase (ALK) protein overexpression in IHC, as reported in up to 45% of cases. [15]. ALK rearrangement is seen mainly in pediatric and younger age groups and is uncommon in adults above 40 years [16]. ALK positivity was also associated with a higher recurrence and less chance of distant metastasis [17]. In our case, atypical oval and spindle cells were arranged in intersecting fascicles, and the cells were ALK-negative on IHC staining. Myofibroblast cells in IMT stain positive for alpha-smooth muscle antigen (SMA), vimentin, and fibronectin, but stains negative for desmin and caldesmon [18]. This helps in differentiating IMT from fibrosarcoma that stains positive for both desmin and caldesmon. Our literature review has shown that 88% of cases stained positive for SMA and none with caldesmon. In our patient, tumor cells stained positive for SMA and caldesmon, but negative for desmin.

Intraoperative frozen section examination or preoperative biopsy rarely confirms the diagnosis due to vast inflammatory infiltrates in the lesion. A recent study has reported a case of pancreatic IMT that was diagnosed by endoscopic ultrasound-guided biopsy [6]. But more studies are needed to confirm the accuracy of endoscopic ultrasound (EUS) and biopsy. Hence, at present, surgical resection remains the treatment of choice both for confirmation of diagnosis and to relieve the symptoms. A rare instance of spontaneous resolution of pancreatic IMT has been reported [6]. The role of chemotherapeutic agents in IMT is yet not established and has mostly been tried in unresectable cases [19]. Recently, few studies have reported successful management of ALK (anaplastic lymphoma tyrosine kinase) positive IMTs with Crizotinib (an ALK inhibitor) [20].

Incomplete resection is one of the factors responsible for tumor recurrence. The prognosis of IMT is generally good with complete surgical resection. In a short follow-up of 3 months, no recurrence or operative complication was encountered in our patient.

## Conclusions

Pancreatic IMTs are rare neoplasms with unpredictable clinical behavior. Clinical and radiological features are usually nonspecific, resulting in preoperative diagnostic dilemmas. The diagnosis ultimately depends on histopathological examination and immunohistochemical studies. Complete resection, which may occasionally require a multi-visceral resection, is the mainstay of treatment. Further research is required in this field to device newer investigation for accurate diagnosis and better outcomes.
